# Supporting the technocracy of artificial intelligence: Data from a representative Spanish sample

**DOI:** 10.1016/j.dib.2025.111637

**Published:** 2025-05-13

**Authors:** Eva Moreno-Bella, Marcos Dono, Miguel Moya

**Affiliations:** a Department of Social and Organizational Psychology, National University of Distance Education (UNED), Madrid, Spain; b Mind, Brain and Behavior Research Center (CIMCYC), University of Granada, Granada, Spain; c Institute of Psychology (IPsiUS), Universidade de Santiago de Compostela, Santiago de Compostela, Spain; d Department of Social Psychology, University of Granada, Granada, Spain

**Keywords:** Artificial intelligence, Technocracy, Socioeconomic status, Political psychology, Economic factors, Open access

## Abstract

The recent development and improvement of generative AI have significantly influenced the lives of all citizens in developed countries. However, this technological advancement is occurring in a context of economic distress and political uncertainty with the rise of the political extremism. In this article we present a dataset collected for examining social and political attitudes of the Spanish population. We obtained participants’ responses through a research company using an online survey. The sample was stratified by quotas following the distribution of the Spanish population. All participants received an economic compensation for their participation. The dataset featured in this article contains data of 1,541 participants on support for technocracy of artificial intelligence (AI), subjective socioeconomic status, political orientation, and other related variables such as financial threat, financial scarcity, perceived socioeconomic decline, perceived economic inequality, and relative deprivation. The survey also collected sociodemographic data. Importantly, these data were collected during a period of political elections in some regions of Spain. This is the first dataset on support for technocracy of AI and social and political attitudes that may shape it in current societies. It is proposed to consider this dataset as a help to clarify and examine the relationship between the perception of economic situation and support for an AI technocracy. This data publication could offer new perspectives for addressing current political and economic challenges, proposing solutions based on data and the potential factors that foster trust in AI as a part of government.

## Graphical abstract

**Figure:**
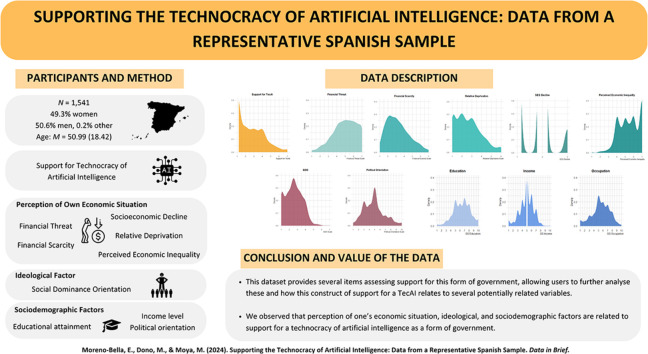

Specifications Table

**Table:** 

Subject	*Social and Personality Psychology / Political Sciences*
Specific subject area	*Social Psychology / Political Psychology*
Type of data	Table, Figure, Data, Raw
Data collection	The field work was carried out by company NETQUEST between 05/24/2023 and 06/01/2023. 1,541 interviews were carried out through the Netquest online panel, using exclusively the panel sample (NOT contracting an external sample). The items included in the questionnaire were selected either from scales and measures already validated or developed by the authors themselves.
Data source location	*Country: Spain*
Data accessibility	Repository name: Open Science Framework Data identification number: https://osf.io/j8w5h/ Direct URL to data: https://osf.io/j8w5h/?view_only=6be7228a94b44b2487328005c6411ffa Instructions for accessing these data: For editors and reviewers to access the data, they may use the link provided above, which is available exclusively for peer review and ensures anonymity.
Related research article	

## 1 Value of the Data

• The present article provides information about the support of Spanish participants to the idea of a technocracy led by Artificial Intelligence (TecAI).
• The appended dataset provides several items assessing support for this form of government, allowing users to further analyse these and how this construct of support for a TecAI relates to several potentially related variables like Social Dominance Orientation, socioeconomic status, perception of financial risks or status decline, and political ideology, among others.
• The database can be used to inform novel research on an innovative topic such as the integration of Artificial Intelligence in politics. Different uses comprise creating validating scales about perceptions of AI in politics or studying predictors of support for such uses.
• This data can also be of use to stakeholders and policymakers as an assessment of political discontent and the current status of acceptance of the integration of AI into politics.
• Considering the exponential growth in AI technologies and their relevance, as well as their increasing implementation in several areas of daily life, we argue that the integration of AI into politics is a key question for the near future in the Social Sciences.
• Secondarily, it can also be used to inform on the relationship between several socioeconomic indicators and economic and status-related perceptions.

## 2 Background

Artificial Intelligence (AI) is a groundbreaking technology transforming human life across numerous fields [1]. Its capacity to learn and develop complex models from vast data enables AI to outperform human capabilities [2]. However, the rapid spread of AI, often without full regard for its legal or psychological impacts, raises challenges, especially regarding decision-making [3] and potential overreliance [4]—issues that become particularly pressing in politics, where decisions shape entire societies. Though politics is susceptible to AI integration, little research exists on public views of AI as a political agent. We aim to explore potential overreliance on AI in politics, potentially leading to support for AI technocracy (TecAI). We examined factors that might drive support for placing political responsibility in AI, drawing on research into political extremism and unconventional political choices that challenge the current system (e.g., [[5], [6], [7], [8]]). The dataset sheds light on psychological and ideological variables, such as economic hardship, that could foster support for AI technocracy. Economic challenges like inequality and deprivation can push people toward extreme political options [9]. Additionally, individuals with high Social Dominance Orientation (SDO) often prefer more extreme politics [10]. This data offers insights into AI-based governance support.

## 3 Data Description

The dataset presents raw data collected to analyse support for TecAI. It includes the sociodemographic data of the sample (e.g., gender, age). It also encompasses variables related to economic factor such as subjective socioeconomic status, (i.e., subjective position in a social ladder based on a) education, b) income, and c) occupation), financial threat, financial scarcity, perceived socioeconomic decline, relative deprivation, perceived economic inequality, social dominance orientation, and political orientation. See the Codebook document for an overview of the measured variables, their corresponding items, and sources. All variables in the document are presented in order of appearance in the survey.

**Fig. 1:**
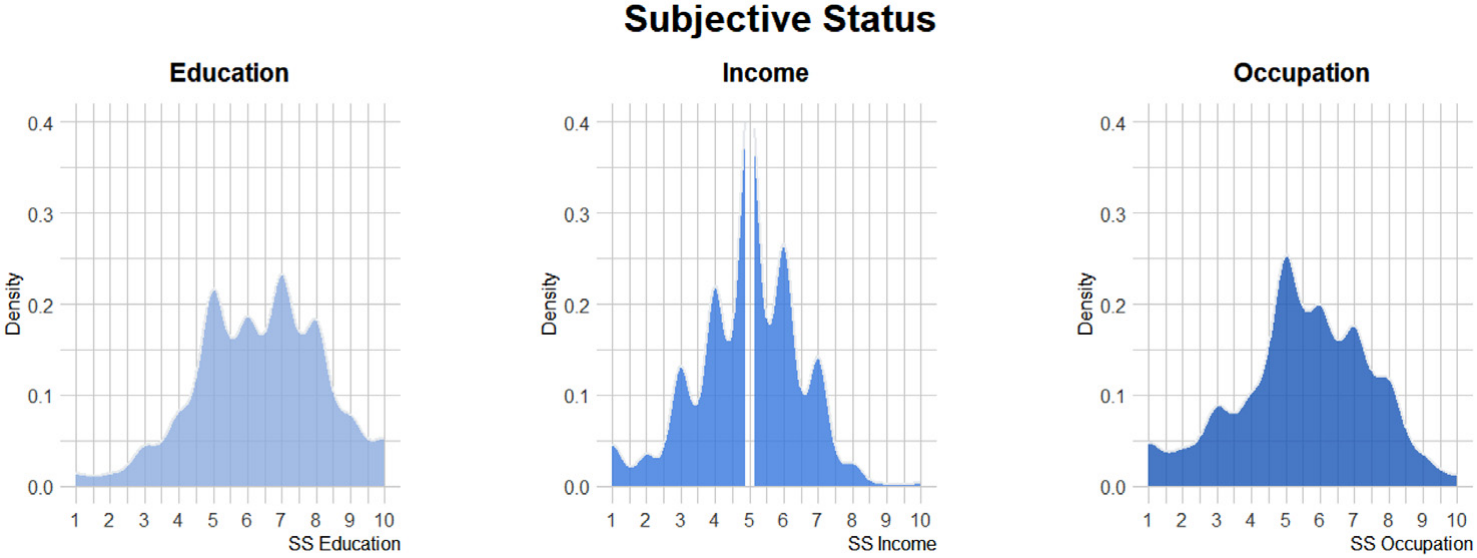
Density of Responses of the Studied Sample in Subjective Status in Relation to Education, Income, and Occupation. *Note.* SS = Subjective Status.

**Fig. 2:**
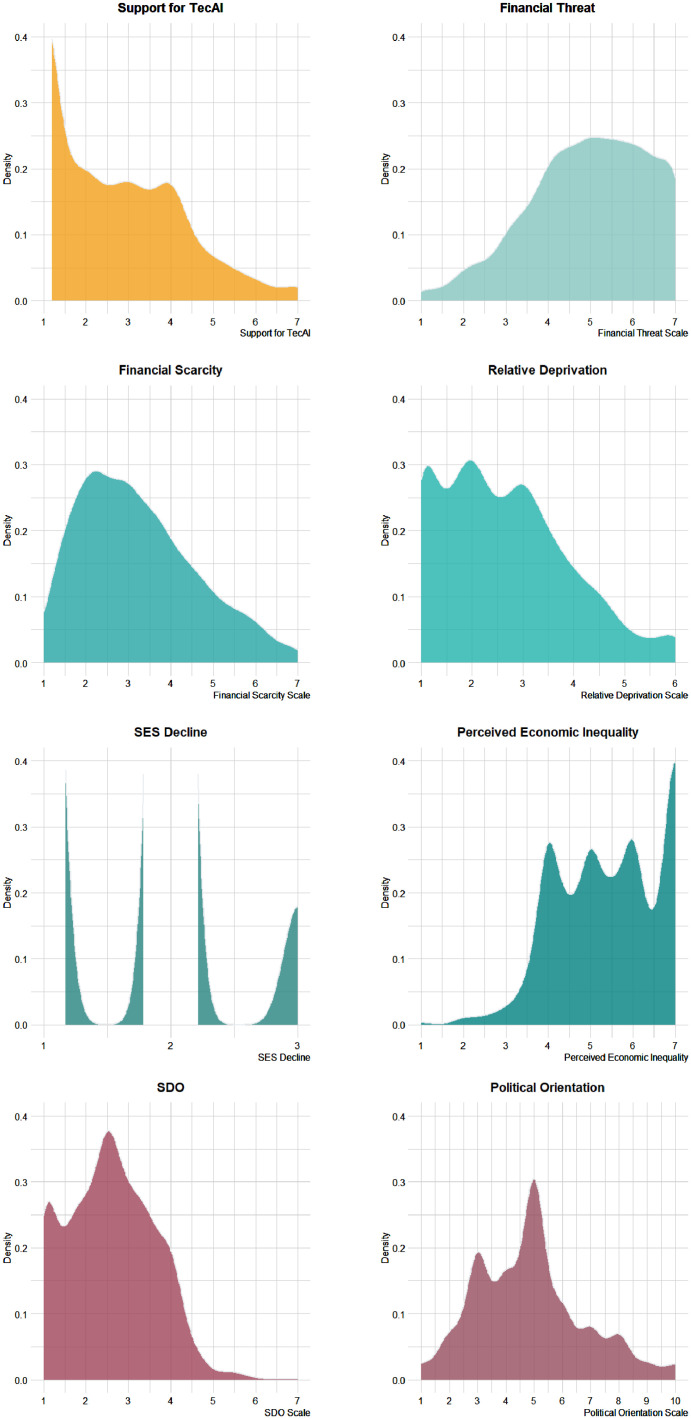
Density of Responses of the Studied Sample in Support for TecAI, Financial Threat, Financial Scarcity, Perceived Socioeconomic Decline, Relative Deprivation, Perceived Economic Inequality, SDO, and Political Orientation. *Note*. TecAI = Technocracy of Artificial Intelligence; SES = Socioeconomic Status; SDO = Social Dominance Orientation.

Density of responses of subjective status in education, income, and occupation are represented in Fig. 1. Representation of response density of support for TecAI, economic-related variables, SDO, and political orientation is available in Fig. 2.

## 4 Experimental Design, Materials and Methods

The study was a cross-sectional design carried out by a group of experts in social psychology and administered online. The main goal of the design was twofold: to perform an initial assessment on the potential reliability of items measuring TecAI and to examine the associations that may exist between perceptions of inequality (objective and relative) and being in favour of using AI as a governing tool. To carry out the study, we contacted to an external online survey research company (i.e., NETQUEST). This company laid out the study in the platform NICEQUEST based on the design of the researchers. The sample size was planned based on economic resources availability (Lakens, 2022). The sample was stratified by quotas following the distribution of the Spanish population based on social class by sex age and region of residence. Social class indicator was calculated by the estimated monthly household income by the company (see Supplementary Materials in OSF). The final sample consisted of 1,541 participants (49.3% women, 50.6% men, 0.2% other; *M_Age_* = 50.99, *SD_Age_* = 18.42). Most of them has 2^nd^ Grade (2^nd^ Cycle, 39.5%) and a mean of income level of 2263.74€ (*SD* = 1687.46). Refer to Table 1 for the regional distribution of participants. A sensitivity analysis revealed that, at power (1-β) = .95, our sample allowed us to find a correlation effect as small as *r =* .08.

**Table 1:** Regional distribution of the studied sample.

Regions	N	Percentage
Northeast / Catalonia and Balearic Islands	196	12.7%
Levante	234	15.2%
South / Andalusia	289	18.8%
Center	144	9.3%
Northwest	142	9.2%
North Central	137	8.9%
Canary Islands	68	4.4%
AMB (Barcelona Metropolitan Area)	131	8.5%
AMM (Madrid Metropolitan Area)	200	13%
**Total of Participants**	1,541	100%

To perform the study, the company contacted participants through their -previously voluntarily provided- email addresses. Participants were provided with the information of the study (e.g., research aim, duration). Before responding to the questionnaire, participants were informed about the confidentiality and anonymity of their responses and gave their consent to participate. This was a requirement for participants when collaborating with NETQUEST. They first completed their sociodemographic information, such as gender, age, and participants’ educational attainment. Later, they completed the entire survey (see Supplementary Materials for the full scales). All the participants received an economic compensation for their participation in our study. Data collection started the 24th of May 2024 and ended 1st of June 2024 in all the regions of Spain (see Table 1 to revise the regions classification). Notably, this period coincided with elections in some regions of Spain; therefore, the leaders of political parties in some regions were actively campaigning, and the national news was providing daily updates on the political situation. Thus, we argue this was a particularly interesting time to examine preferences about the use of AI in politics.

### 4.1 Measures

*Sociodemographic measures.* Participants were asked to provide information on a series of sociodemographic indicators including gender, age, educational level, parental situation (whether their parents are still alive), parent’s educational level, their income level and the number of family members who may be under their care (family members in the household under 14 years old).

*Political orientation.* It was assessed via a single-item measure in which participants had to self-place themselves in an ideological continuum ranging from 1 (*Extreme left-wing*) to 10 (*Extreme right-wing*).

*Perceived financial threat*. In order to measure the degree of *perceived financial threat* we adapted a previous instrument by [11] by using 4 items (e.g. “To what extent are you worried about your personal economic situation?”) arranged on a Likert scale (1= *Not at all*, 7 = *Very Much*). Reliability was excellent (α = .86).

*Perceived Socioeconomic Status Decline*. Participants were asked to evaluate changes in their and their family’s socioeconomic status over recent years through the following statement: “During the past few years, and thinking about your and your family’s economic situation, please choose the option that best describes your situation.” following [12]. Responses were recorded on a three-point scale ("Our socioeconomic status has worsened, and we have moved from being in one social class to a lower one." coded as 1, “Our socioeconomic status remains the same.” coded as 2, and “Our socioeconomic status has improved”, and “We have moved from being in one social class to a higher one.” coded as 3).

*Financial scarcity*. The perception of being in a situation of financial scarcity was measured by using a 12-item, 7-point Likert scaled (1= *Totally disagree* to 7 = *Totally agree*) instrument adapted from [13] (e.g. “*I often don’t have enough money*.”; α = .91). The composite variable was the average of the scores on the 12 items.

*Subjective Status*. We measured subjective status in three dimensions (income, education, occupation). Regardless of the domain, the instrument was identical, adapted from [14]. It consists in a single-item measure that uses a graphical representation of a ladder and asks participants to situate themselves in such ladder by imagining it represents the stratification of society in the corresponding dimension (i.e. income). Thus, they choose to place themselves in one of the rungs of the ladder, from the lowest (0) to the highest (10).

*Relative deprivation*. We also measured relative deprivation [15,16] with the Spanish version of the original instrument [17]. To this end we used 6 items (e.g. “I feel disadvantaged when I think about what I have compared to other people like me.”) arranged in a 6-point Likert scaling (1 = *Totally disagree*, 6 = *Totally agree*). The 6 items were averaged to create the composite variable. The reliability of the instrument turned out to be excellent (α = .91).

*Social Dominance Orientation.* It was measured by using the measure by [8] consisting in 4 items (e.g. “When setting priorities, we should consider all groups.”) ranging from 1 (totally disagree) to 7 (totally agree). This measure includes two reverse-coded items (see the Codebook document). After reversing these two items, the composite variable was created by calculating the mean of the four items. Despite its previous validation, the scale showed poor reliability (α = .55).

*TecAI.* In order to measure TecAI we used a 3-item, 7-point Likert scaled (1= *Totally disagree* to 7 = *Totally agree*) measure based on an instrument previously used in Dono & Moreno-Bella [18] (e.g. *“I believe that Parliament would function better if important decisions were made by artificial intelligence*”). We averaged the score of all the items. The reliability index of the scale was good (α = .82).

*Perceived Economic Inequality*. Lastly, we also assessed the degree in which participants perceived Spanish society to be unequal by asking two complimentary questions on the matter (“To what extent do you believe that distribution of resources in Spain is UNEQUAL [EQUAL]?”) [19] in 7-point Likert scale items (1 = *Not at all*, 7 = *Completely*). The question about the equality of the distribution was a reverse-coded item. Therefore, we reversed the coding for that item and calculated the mean of the two items (*rho_Spearman−Brown_* = .65, *p* < .001). Higher scores indicate higher perceived economic inequality.

## Limitations

The limitations of this dataset stem from the fact that the data are part of a larger database originally designed for other specific research purposes. While it would be interesting to relate support for AI technocracy with other psychosocial variables, this is not possible due to the constraints imposed by the objectives of each subproject.

Additionally, it is important to note that the full scales could not be utilized to assess certain constructs due to space limitations in the larger survey. Therefore, it is recommended to execute further research using the corresponding complete scales to better/more in-depth examine the potential relations between the variables.

## Ethics Statement

All procedures performed in this research involving human participants followed the ethical standards of the Vicerectory of Research and Scientific Policy of the University of Granada (Reference Number: 1856/CEIH/2020), and it is in accordance with the 1964 Declaration of Helsinki. All participants were informed in writing about the objectives of the study and the voluntariness of participation.

## Credit Authors Statement

**Eva Moreno-Bella:** Conceptualization, methodology, formal analysis, investigation, writing – original draft, writing – review & editing, visualization. **Marco Dono**: Conceptualization, writing - review & editing**. Miguel Moya**: investigation, writing – review & editing, project administration, funding acquisition.

## Data Availability

Open Science FrameworkTECAI (Original data)

## Appendix Supplementary materials

Image, application 1
