# Supplementary material for: Supporting the technocracy of artificial intelligence: Data from a representative Spanish sample
Source: Data Brief. 2025 May 13;60:111637. doi: 10.1016/j.dib.2025.111637 (PMC12151244; doi:10.1016/j.dib.2025.111637)
Supplement: Supplementary file 1 [file mmc1.pdf]

Supplementary Materials pertaining to the article

"Supporting the Technocracy of Artificial Intelligence: Data from a Representative Spanish Sample"

The document provides details on the new socioeconomic classification system to be used in the EGM (Estudio General de Medios) starting from the first wave of 2015. This new system replaces the previous social class index established in 1988. The document describes the new variable along with the questions necessary for its calculation.

Key Points:

1. New Classification System:

- The new classification is based on the estimation of household income, which is calculated using a specific formula.

- The formula takes into account household size and the number of individuals with income, adjusting these factors to estimate the household's income level.

Group = Group_i

Main Supporter's Activity = Activity_j

Matrix = Matrix Row_m Column_n

Income Estimate = Group + Activity + Matrix

Meaning:

Group 1 = 1,1380	Group 7 = 84
Group 2 = 1,113	Group 8 = 0
Group 3 = 710	Actividad 1 (trabaja) 3,028
Group 4 =474	Actividad 2 (jubilado) = 2,650
Group 5 = 355	Actividad 3 (parado) = 2.384
Group 6 = 190	Actividad 4 (inactive) = 2,705

Household size	Number of individuals with income: 0/1	2	3	4+
1	-2136	-	-	-
2	-1998	-1422	-	-
3	-1995	-1305	-836	-
4	-1922	-1238	-681	-182
5	-1957	-1309	-788	99
6+	-1965	-1421	-905	0

GRUPO	MENOS DE PRIMARIOS	PRIMARIOS INCOMP.	1er GRADO	2do GRADO 1er CICLO	2do GRADO 2do CICLO	3er GRADO MEDIO	3er GRADO SUPERIOR	RESTO
Director gran empresa	2	2	2	2	1	1	1	2
Comerciante 6/+ empleados	2	2	2	2	2	1	1	2
Mando superior	2	2	2	2	2	2	1	2
Agricultor 6/+ empleados	3	2	2	2	2	2	2	2
Director pequeña empresa	3	3	2	2	2	2	2	2
Profesional liberal	4	3	3	2	2	2	2	3
Mando intermedio	4	4	3	3	2	2	2	3
Comerciante 1-5 empleados	4	4	4	3	3	2	2	3
Representante	4	4	4	3	3	3	2	3
Administrativo	4	4	4	4	3	3	3	4
Capataces	4	4	4	4	4	3	3	4
Comerciante Sin empleados	6	4	4	4	4	4	3	4
Agricultor 1-5 empleados	6	6	4	4	4	4	4	5
Miembro cooperativa no agraria	6	6	6	4	4	4	4	5
Subalternos	6	6	6	6	4	4	4	5
Obrero especializado	6	6	6	5	5	4	4	5
Trabajador manual	6	6	6	6	6	4	4	5
Vendedores	7	6	6	6	6	6	4	6
Agricultor en cooperativa	7	7	6	6	6	6	6	6
Obreros no especializados	7	7	7	6	6	6	6	6
Agricultor Sin empleados	8	7	7	6	6	6	6	7
RESTO	8	8	7	7	6	6	6	7
Otro no cualificado	8	8	7	7	7	6	6	7
Jornaleros	8	8	8	7	7	6	6	7

3. Income and Socioeconomic Status:

- The table details how different professional and educational levels are coded to classify the socioeconomic status of the primary breadwinner in the household.

- This information is crucial for assigning the household to one of the seven income brackets defined by the new classification system. For more information, contact the company NETQUEST.

4. Socioeconomic Classification Brackets:

- Households are categorized into seven brackets based on their estimated income:
    - A1: More than 3005
    - A2: From 2452 to 3005
    - B: From 2146 to 2451
    - C: From 1603 to 2145
    - D: From 1313 to 1602
    - E1: From 745 to 1312
    - E2: Up to 744

This new classification system aims to provide a more accurate and current reflection of the socioeconomic status of households, based on updated income data and relevant social factors.
